# Effectiveness of Acetazolamide in Patients With Heart Failure: A Systematic Review and Meta-Analysis

**DOI:** 10.7759/cureus.75778

**Published:** 2024-12-16

**Authors:** Sanjay Eda, Mandeep Kaur, Mian M Rehman, Sindhuja Sompalli, Keron Blair, Sandipkumar S Chaudhari, Calvin R Wei, Danish Allahwala

**Affiliations:** 1 Medicine, MNR Medical College and Hospital, Sangareddy, IND; 2 Hospital Medicine, Hospital Corporation of America (HCA) Capital Regional Medical Center, Tallahassee, USA; 3 Cardiology, Combined Military Hospital (CMH) Lahore Medical College and Institute of Dentistry, Lahore, PAK; 4 Internal Medicine, JSS Medical College, Mysuru, IND; 5 Medicine, American International School of Medicine, Georgetown, GUY; 6 Cardiothoracic Surgery, University of Alabama at Birmingham, Birmingham, USA; 7 Family Medicine, University of North Dakota School of Medicine and Health Sciences, Fargo, USA; 8 Research and Development, Shing Huei Group, Taipei, TWN; 9 Nephrology, Fatima Memorial Hospital, Karachi, PAK

**Keywords:** acetazolamide, diuresis, heart failure, natreuresis, systematic review and meta analysis

## Abstract

This systematic review and meta-analysis evaluated the efficacy and safety of acetazolamide as an adjunctive diuretic therapy in acute heart failure (AHF) patients. A comprehensive literature search was conducted across multiple electronic databases, including PubMed, Embase, Cochrane Library, and Scopus, identifying seven studies (five randomized controlled trials and two observational studies) that met the eligibility criteria. The analysis revealed that acetazolamide significantly enhanced mean natriuresis (mean differences (MD) 52.72 mmol, 95% confidence interval (CI: 15.52 to 89.92) and mean diuresis (MD 432.88 mmol, 95% CI: 205.82 to 659.93) compared to control groups. However, no significant difference was observed in all-cause mortality between patients receiving acetazolamide and those who did not (relative risks (RR): 1.23, 95% CI: 0.86 to 1.76, p-value: 0.25). While high heterogeneity was reported in natriuresis outcomes (I^2^: 90%), diuresis results showed no heterogeneity (I^2^: 0%). These findings suggest that acetazolamide effectively enhances fluid removal when used in combination with standard loop diuretics, supporting its role as adjunctive therapy in AHF management. However, limitations, including the small number of studies and inclusion of both RCTs and observational studies, indicate the need for further large-scale trials to better understand acetazolamide's impact on long-term outcomes and identify specific patient populations who may benefit most from this therapy.

## Introduction and background

Acute heart failure (AHF) represents a substantial clinical challenge due to its high morbidity, mortality, and frequent hospital readmissions [1]. Patients with AHF often present with significant congestion due to fluid overload, a key driver of symptoms such as dyspnea, peripheral edema, and overall worsening of cardiac function [2]. The management of fluid overload in AHF is critical to improving patient outcomes, with diuretics serving as a cornerstone of therapy. Intravenous loop diuretics, such as furosemide, are frequently employed as first-line agents to relieve congestion by promoting renal sodium and water excretion [3]. However, the response to loop diuretics can be variable and limited by factors such as renal dysfunction and diuretic resistance, which is often observed in patients with AHF. In such cases, adjunctive diuretic therapies may be necessary to enhance decongestion and optimize fluid balance [4,5]. 

Acetazolamide, a carbonic anhydrase inhibitor, has emerged as a promising adjunctive agent for enhancing diuresis in AHF patients who exhibit diuretic resistance [6]. By inhibiting carbonic anhydrase in the proximal renal tubules, acetazolamide reduces the reabsorption of sodium bicarbonate, which in turn increases sodium and water excretion [7]. This mechanism of action is complementary to that of loop diuretics, which primarily target the loop of Henle. Thus acetazolamide has the potential to augment diuresis when loop diuretics alone are insufficient [6]. Acetazolamide has a well-established role in the management of conditions such as glaucoma, altitude sickness, and metabolic alkalosis, but its use in AHF as an adjunctive diuretic is less well-defined [8]. Recent studies have sought to explore its effectiveness in AHF, suggesting that acetazolamide may provide added benefits in terms of fluid removal and symptom relief [9-12]. 

While preliminary data on the use of acetazolamide in AHF have been encouraging, a comprehensive understanding of its efficacy and safety as an adjunctive diuretic therapy remains lacking. This gap underscores the need for a systematic review and meta-analysis to synthesize available evidence and provide clinicians with clear guidance on the role of acetazolamide in the management of AHF. Such an analysis is particularly pertinent given the ongoing challenge of managing diuretic resistance and optimizing fluid balance in AHF patients, who often face limited therapeutic options beyond loop diuretics [13,14]. 

The purpose of this meta-analysis is to evaluate the efficacy and safety of acetazolamide as an adjunctive diuretic therapy for patients with AHF. By pooling data from relevant clinical trials, this study aims to assess the impact of acetazolamide on key outcomes such as diuresis, fluid balance, symptom relief, hospital readmission rates, and mortality. Additionally, this analysis will examine the incidence of adverse events associated with acetazolamide, providing insights into its safety profile in this vulnerable patient population. Through this systematic review and meta-analysis, we hope to clarify the potential role of acetazolamide in enhancing diuretic response and improving clinical outcomes for patients with AHF. 

## Review

Methodology 

This systematic review and meta-analysis were conducted to assess the efficacy and safety of acetazolamide as an adjunctive diuretic therapy in patients with AHF. The methodology adhered to the Preferred Reporting Items for Systematic Reviews and Meta-Analyses (PRISMA) guidelines to ensure rigorous and transparent reporting. 

Eligibility Criteria 

We defined inclusion criteria prior to the literature search to select relevant studies. Studies were eligible if they met the following criteria: (1) randomized controlled trials (RCTs) or observational studies that evaluated acetazolamide as an adjunct to standard diuretic therapy in patients diagnosed with AHF; (2) reported on clinical outcomes such as diuresis, fluid balance, symptom relief, hospital readmission rates, or mortality; and (3) provided data on adverse events associated with acetazolamide. We excluded non-comparative studies, reviews, case reports, animal studies, or studies that did not focus on AHF. 

Search Strategy 

A comprehensive literature search was conducted across multiple electronic databases, including PubMed, Embase, the Cochrane Library, and Scopus, to identify studies published up to 10 November 2024. The search was designed to capture all relevant studies by using a combination of keywords and medical subject headings (MeSH) terms related to “acetazolamide,” “acute heart failure,” and “diuretics.” No language restrictions were applied. Additionally, reference lists of included studies were manually screened to identify any potentially relevant articles not captured in the initial database search. 

Study Selection and Data Extraction 

Two independent reviewers screened the titles and abstracts of all identified articles to assess their relevance based on the eligibility criteria. Full texts of potentially relevant studies were then evaluated in detail. Any disagreements between reviewers were resolved through discussion or by consulting a third reviewer. Data extraction was performed independently by two reviewers using a standardized form. Extracted data included study characteristics (e.g., study design, sample size, and duration), patient demographics, details of acetazolamide administration, and reported clinical outcomes. The primary outcomes of interest were diuresis, fluid balance, and symptom relief, while secondary outcomes included hospital readmission rates, mortality, and adverse events. 

Risk of Bias Assessment 

The risk of bias for each included study was assessed independently by two reviewers using the Cochrane Risk of Bias tool for RCTs and the Newcastle-Ottawa Scale for observational studies. Studies were evaluated for potential biases related to selection, performance, detection, attrition, and reporting. Any discrepancies in the assessment were resolved through discussion. 

Data Synthesis and Statistical Analysis 

We conducted a meta-analysis using a random-effects model, assuming variability across studies. The pooled estimates were calculated for the primary and secondary outcomes, and results were presented as relative risks (RR) or mean differences (MD) with corresponding 95% confidence intervals (CIs). Statistical heterogeneity was assessed using the I² statistic, with values above 50% indicating substantial heterogeneity. All analyses were performed using RevMan (The Cochrane Collaboration, London, GBR) with a significance level set at p < 0.05.

Results 

**Figure 1:**
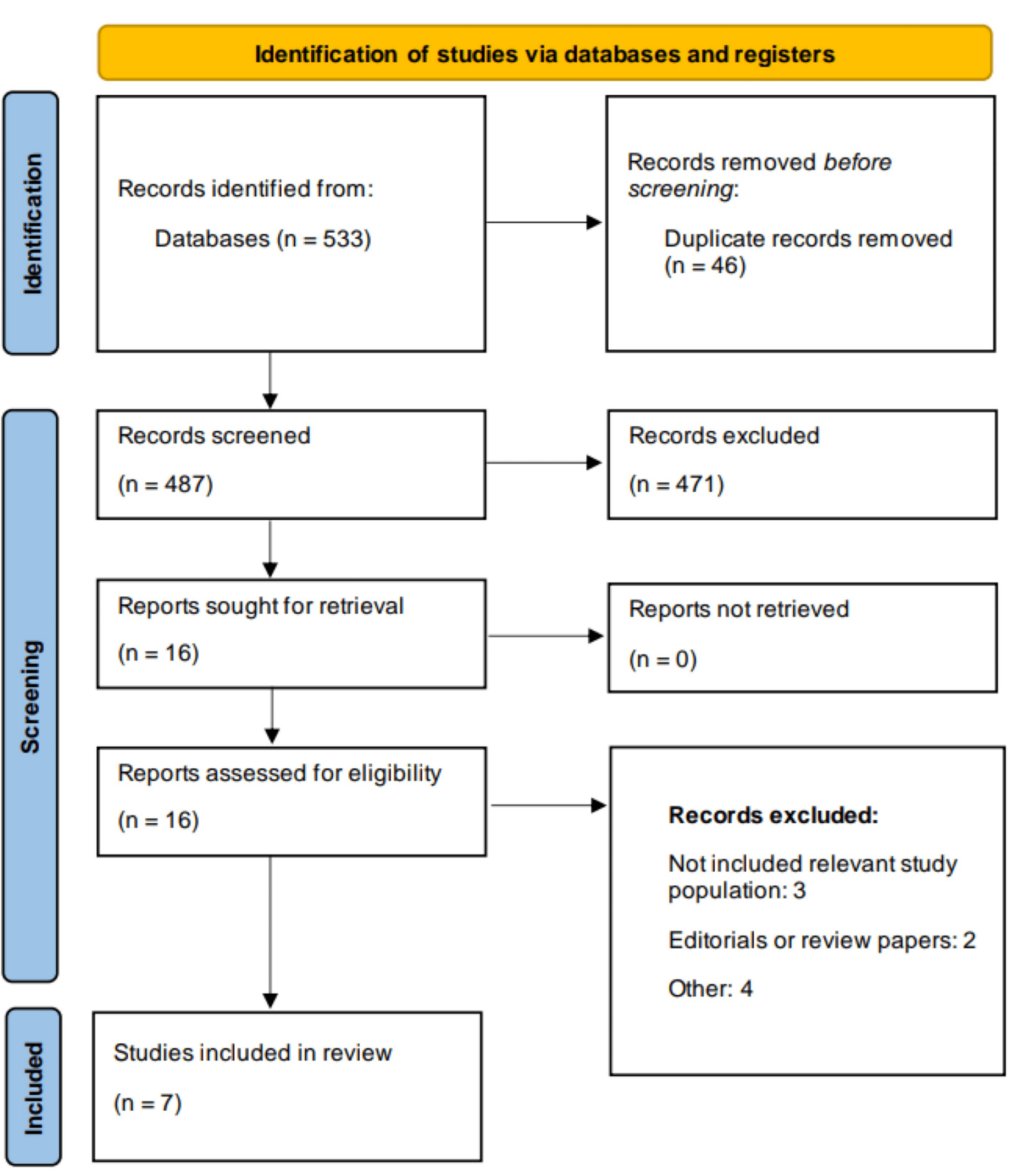
Study selection process (PRISMA flowchart) PRISMA: Preferred Reporting Items for Systematic Reviews and Meta-Analyses

**Figure 2:**
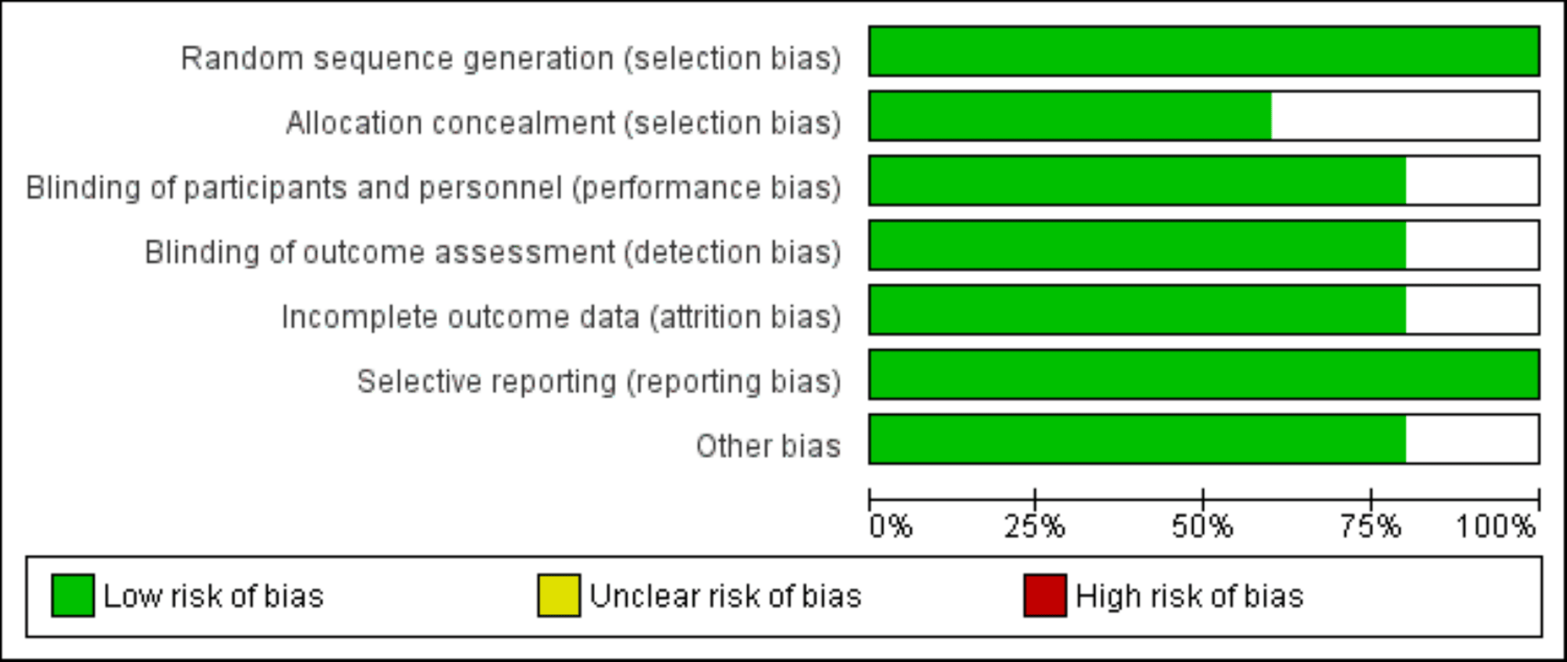
Risk of bias graph of included RCTs RCTs: Randomized-control trials

Through initial database searching, 533 studies were obtained. After removing 46 duplicate records, initial screening was performed based on predefined inclusion and exclusion criteria. Through initial screening, we found 16 studies to be eligible for full-text screening. In the end, seven studies met the eligibility criteria and were included in this meta-analysis. The PRISMA flowchart describing the process of the search and study selection is shown in Figure 1. There were five RCTs and two observational studies. Table 1 shows the characteristics of the included studies. Figure 2 presents a risk of bias assessment of included RCTs.

**Table 1:** Included studies details NR: Not reported; RCT: Randomized-control trial

Author ID	Study design	Setting	Group	Sample size	Follow-up	Dose of acetazolamide	Mean age (years)	Males (n)
Imeila et al., 2017 [10]	RCT	Single center	Acetazolamide	10	4 days	250–500 mg daily	73	8
			Without acetazolamide	10			71.2	9
Kosiorek et al., 2023 [14]	RCT	Single center	Acetazolamide	31	3 days	250 mg	69	22
			Without acetazolamide	30			68	21
Martin et al., 2023 [15]	RCT	Single center	Acetazolamide	26	NR	NR	77	20
			Without acetazolamide	29			77	16
Mercado et al., 2024 [16]	Prospective observational	Single center	Acetazolamide	28	3 months	500 mg	78	18
			Without acetazolamide	30			85	12
Mullens et al., 2022 [11]	RCT	Multicenter	Acetazolamide	260	3 months	500 mg	77.9	170
			Without acetazolamide	259			78.5	155
Verbrugge et al., 2015 [17]	Prospective observational	Single center	Acetazolamide	37	NR	250 mg	NR	NR
			Without acetazolamide	17				
Verbrugge et al., 2018 [12]	RCT	Multicenter	Acetazolamide	18	34 months	250-500 mg daily	81	11
			Without acetazolamide	16			78	12

*All-cause Mortality* 

**Figure 3:**
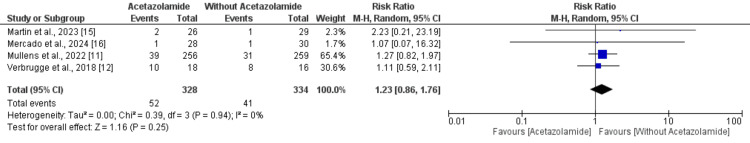
Effect of acetazolamide on all-cause mortality M-H: Mantel-Haenszel statistic. References [11-12, 15-16].

Four studies determined the impact of acetazolamide on all-cause mortality, and the results are presented in Figure 3. The pooled incidence of all-cause mortality was 14.19%, and the risk of all-cause mortality was not significantly different between patients who received acetazolamide and patients who did not receive acetazolamide (RR: 1.23, 95% CI: 0.86 to 1.76, p-value: 0.25). No heterogeneity was reported, as shown by the I^2^ value of 0%. 

*Natriuresis and Diuresis* 

**Figure 4:**
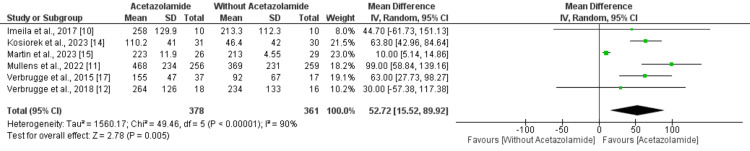
Effect of acetazolamide on natriuresis IV: Implied volatility. References [10-12,14,15,17].

**Figure 5:**
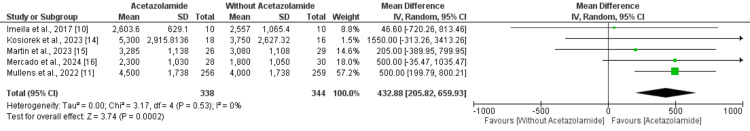
Effect of acetazolamide on diuresis IV: Implied volatility. References [10,11,14-16]

A combined analysis of six studies showed that compared to the control group acetazolamide significantly increased mean natriuresis (MD 52.72 mmol, 95% CI: 15.52 to 89.92) as shown in Figure 4. High heterogeneity was reported among the study results (I^2^: 90%). A combined analysis of five studies showed that, compared to patients without acetazolamide, patients with acetazolamide have significantly increased mean diuresis (MD 432.88 mmol, 95% CI: 205.82 to 659.93) as shown in Figure 5. No heterogeneity was reported among the study results (I^2^: 0%).

Discussion 

In this meta-analysis involving seven studies, we found that acetazolamide is significantly associated with an increase in mean diuresis and natriuresis in patients with heart failure. However, no significant difference was reported between the two groups in risk of all-cause mortality. Similar findings were found in the meta-analysis performed by Malik et al. [13] that included three RCTs. The study reported that compared to patients in the control group, mean natriuresis and diuresis was significantly higher in acetazolamide patients. The consistency of these findings across multiple studies strengthens the evidence supporting the use of acetazolamide as an adjunctive therapy in AHF management. 

The significant increase in natriuresis and diuresis observed in this study supports the proposed mechanism of action of acetazolamide. As a carbonic anhydrase inhibitor, acetazolamide acts on the proximal renal tubules to reduce sodium bicarbonate reabsorption, thereby increasing sodium and water excretion [18]. This mechanism complements the action of loop diuretics, which primarily target the loop of Henle, explaining the enhanced diuretic response observed in patients receiving acetazolamide as an adjunctive therapy [19]. 

The lack of a significant difference in all-cause mortality between the acetazolamide and control groups is an important finding that warrants further discussion. While the primary goal of diuretic therapy in AHF is to relieve congestion and improve symptoms, the impact on long-term outcomes such as mortality remains a critical consideration. The absence of a mortality benefit in this meta-analysis suggests that while acetazolamide may effectively enhance fluid removal, its effects on overall survival may be limited. This highlights the complex nature of AHF and the need for comprehensive management strategies that address not only fluid overload but also underlying cardiac dysfunction and comorbidities [20,21]. 

It is important to note the high heterogeneity (I2: 90%) reported among the study results for natriuresis. This heterogeneity may be attributed to variations in study designs, patient populations, dosing regimens, and concomitant therapies across the included studies. Future research should aim to identify factors contributing to this heterogeneity and explore potential subgroups of patients who may derive the greatest benefit from acetazolamide therapy. 

Since the release of the 2021 European Society of Cardiology (ESC) guidelines on heart failure, acetazolamide has been acknowledged as a possible supplementary treatment alongside diuretics. However, its use has been approached with caution due to limited high-quality evidence. The recently updated ESC 2023 guidelines on heart failure have reinforced this recommendation, drawing on findings from the ADVOR trial. Nevertheless, they emphasize the need for additional research [20]. 

This meta-analysis demonstrates that acetazolamide enhances the efficacy of loop diuretics in patients with AHF, as evidenced by significant increases in diuresis and natriuresis. While the effectiveness of loop diuretics has been established as a strong and independent predictor of clinical outcomes in AHF, it remains unclear whether this relationship is causal. The synergistic effect of acetazolamide with loop diuretics suggests a potential for improved fluid management in patients with AHF, though further research is needed to determine its impact on long-term clinical outcomes [17]. 

Study Limitations 

One limitation of this meta-analysis is the relatively small number of included studies, which may limit the generalizability of the findings. Additionally, the inclusion of both RCTs and observational studies introduces potential bias and heterogeneity in the analysis. Future large-scale, well-designed RCTs are needed to further validate these findings and address remaining questions regarding the optimal use of acetazolamide in AHF. The role of acetazolamide in specific subgroups of AHF patients, such as those with preserved versus reduced ejection fraction, varying degrees of renal dysfunction, or different etiologies of heart failure, remains to be elucidated. Subgroup analyses in future studies could help identify patient populations that may derive the greatest benefit from acetazolamide therapy and guide more personalized treatment approaches. Furthermore, the impact of acetazolamide on other important clinical outcomes, such as length of hospital stay, readmission rates, and quality of life measures, should be explored in future research. These endpoints are crucial for assessing the overall effectiveness and cost-effectiveness of acetazolamide as an adjunctive therapy in AHF management. 

## Conclusions

Based on the meta-analysis, acetazolamide demonstrates promising efficacy as an adjunctive therapy in AHF management. The analysis of seven studies revealed significant improvements in both natriuresis and diuresis when acetazolamide was combined with standard loop diuretics. However, there was no significant impact on all-cause mortality between treatment groups. While these findings support acetazolamide's role in enhancing fluid removal, its effect on long-term survival outcomes remains unclear. The high heterogeneity in natriuresis results suggests the need for further investigation to identify optimal patient populations and treatment protocols. Future large-scale randomized controlled trials should focus on specific subgroups, examining outcomes such as hospital readmission rates, length of stay, and quality of life to better define acetazolamide's place in AHF therapy.
